# New insights of growth hormone (GH) actions from tissue-specific GH receptor knockouts in mice

**DOI:** 10.20945/2359-3997000000185

**Published:** 2019-11-01

**Authors:** Edward O. List, Darlene E. Berryman, Elizabeth A. Jensen, Prateek Kulkarni, Savannah McKenna, John J. Kopchick

**Affiliations:** 1 The Edison Biotechnology Institute Ohio University Athens Ohio USA The Edison Biotechnology Institute, Ohio University, Athens, Ohio, USA; 2 The Department of Biomedical Sciences Heritage College of Osteopathic Medicine Ohio University Athens Ohio USA The Department of Biomedical Sciences, Heritage College of Osteopathic Medicine, Ohio University, Athens, Ohio, USA

**Keywords:** Tissue-specific knockout, GHR, GHRKO, conditional knockout, gene disruption

## Abstract

In order to provide new insights into the various activities of GH in specific tissues, recent advances have allowed for the generation of tissue-specific GHR knockout mice. To date, 21 distinct tissue-specific mouse lines have been created and reported in 28 publications. Targeted tissues include liver, muscle, fat, brain, bone, heart, intestine, macrophage, pancreatic beta cells, hematopoietic stem cells, and multi-tissue “global”. In this review, we provide a brief history and description of the 21 tissue-specific GHR knockout mouse lines. Arch Endocrinol Metab. 2019;63(6):557-67

## INTRODUCTION

**Figure 1:**
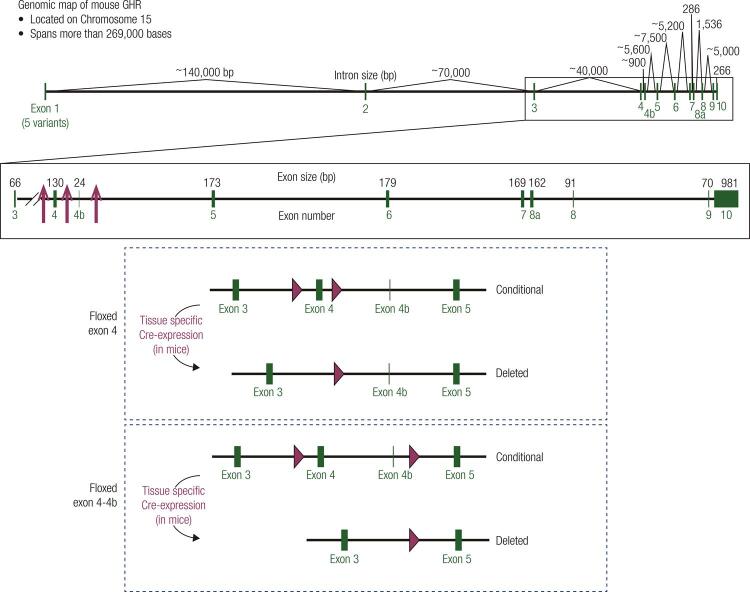
Targeting strategy to generate floxed GHR mice. (Top) Genomic map of the mouse GHR gene. The mouse GHR gene is located on chromosome 15 and spans more than 269,000 bases. Exons are shown in green. The site of integration for the loxP sequences used to generate floxed GHR mice are indicated by red arrows (4-7). (Middle/Bottom) Cartoon representation indicating location of lox P sites for floxed exon 4 strategy used by the Sperling (4), LeRoith (5), and Kopchick (6) laboratories and floxed exon 4-4b strategy used by the Liang laboratory (7).

**Figure 2:**
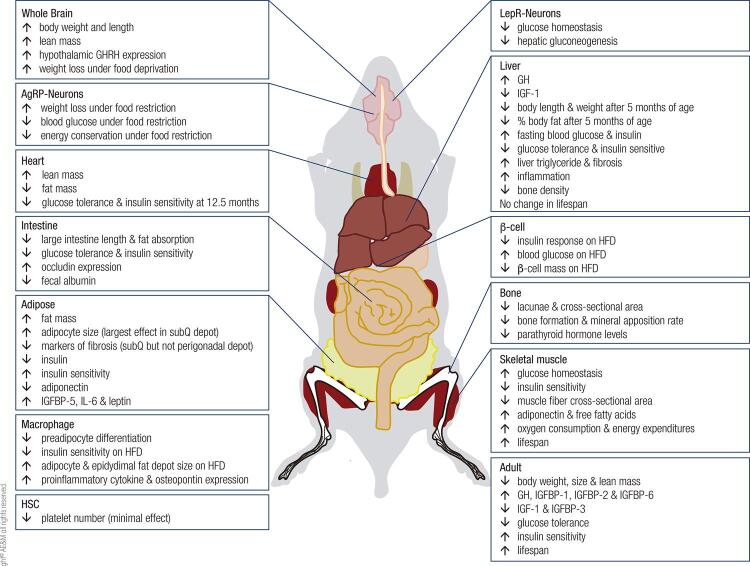
Summary of results from conditional GHR gene disrupted mice.

Growth hormone (GH) is pleiotropic hormone involved in many diverse processes such as growth, adiposity, glucose homeostasis, reproduction, and longevity. To help uncover the various activities of GH, global GH receptor (GHR) gene-disrupted or knock-out (GHRKO) mice were generated more than 20 years ago ( 1 ). Since that original study, GHRKO mice have been used in 128 published studies greatly expanding our knowledge of the GH/IGF-1 axis ( 2 , 3 ). While GHRKO mice have become a useful tool to help uncover the various activities of GH, discerning the direct actions of GH on individual tissues has proven challenging. Accordingly, a number of tissue-specific GHRKO mice have been developed over the last decade to better understand the direct actions of GH in selective tissues. To generate these mice, 4 distinct GHR floxed mouse lines have been generated by 4 different laboratories, the Sperling laboratory in 2009 ( 4 ), the LeRoith laboratory in 2011 ( 5 ), the Kopchick laboratory in 2013 ( 6 ), and the Liang laboratory in 2019 ( 7 ) ( Table 1 and Figure 1 ). These GHR floxed mice have been crossed to transgenic mice containing a Cre-recombinase (Cre) gene preceded by a promoter/enhancer to drive expression in select tissues. When both a tissue-specific Cre mouse line and a GHR floxed mouse line have successfully been crossed to generate a mouse line that is both homozygous floxed and Cre positive, recombination of the floxed GHR takes place resulting in a tissue-specific GHR “knockout” mouse. To date, at least 21 distinct tissue-specific GHR gene disrupted mouse lines have been generated: liver, muscle, fat, brain, bone, heart, intestine, macrophage, pancreatic b cells, hematopoietic stem cells, and multi-tissue “global” ( Table 2 and Figure 2 ). In this review, we will give a brief history of each line followed by results obtained when GHR is disrupted in a given tissue.

**Table 1:** The four floxed GHR mouse lines

Year	Reference	Region of GHR Floxed	Laboratory of	University
2009	Fan et al., 2009 ( 4 )	Exon 4	Mark A. Sperling	University of Pittsburgh School of Medicine, Pittsburg, PA, USA
2011	Wu et al., 2011 ( 5 )	Exon 4	Derek LeRoith	Mount Sinai School of Medicine, New York, NY, USA
2013	List et al., 2013 ( 6 )	Exon 4	John J. Kopchick	Edison Biotechnology Institute, Ohio University, Athens, OH, USA
2019	Fang et al., 2019 ( 7 )	Exon 4 through 4b	Guosheng Liang	University of Texas Southwestern Medical Center, Dallas TX, USA

**Table 2:** Chronological list of conditional GHR gene disrupted mice

Year	Reference	Target Tissue	Inducible	Line Designation	Floxed GHR Line	Cre Line	Stock/Cat. No.
2009	Fan et al., 2009 ( 4 )	Liver	-	GHRLD	Sperling	B6.Cg-Tg(Alb-cre)21Mgn/J	JAX: 003574
2010	Lu et al., 2010 (25)	Macrophage	-	GHRMacD	Sperling	B6.129P2-Lyz2tm1(cre)Ifo/J	JAX: 004781
2010	Mavalli et al., 2010 (33)	Muscle	-	∆GHR	Sperling	Mef-2c-73kCre+/-/Blk	MGI ID: 3583679
2011	Wu et al., 2011 (5)	Beta cell	-	βGHRKO	LeRoith	B6.Cg-Tg(Ins2-cre)25Mgn/J	JAX: 003573
2012	Vijayakumar et al., 2012 (34)	Muscle	-	mGHRKO	LeRoith	B6.FVB(129S4)-Tg(Ckmm-cre)5Khn/J	JAX: 006475
2012	Vijayakumar et al., 2012 (35)	Muscle	-	mGHRKO	LeRoith	B6.FVB(129S4)-Tg(Ckmm-cre)5Khn/J	JAX: 006475
2013	List et al., 2013 (6)	Fat	-	FaGHRKO	Kopchick	B6.Cg-Tg(Fabp4-cre)1Rev/J	JAX: 005069
2013	Vijayakumar et al., 2013 (36)	Muscle	-	mGHRKO	LeRoith	B6.FVB(129S4)-Tg(Ckmm-cre)5Khn/J	JAX: 006475
2013	Li et al., 2013 (10)	Fat	-	FaGHRKO	Kopchick	B6.Cg-Tg(Fabp4-cre)1Rev/J	JAX: 005069
2013	Lu et al., 2013 (26)	Macrophage	-	MacGHR KO†	Sperling	B6.129P2-Lyz2^tm1(cre)Ifo^/J	JAX: 004781
2014	List et al., 2014 (9)	Liver	-	LiGHRKO	Kopchick	B6.Cg-Tg(alb-cre)21Mgn/J	JAX: 003574
2014	Fan et al., 2014 (8)	Liver	-	GHRLD	Sperling	6.Cg-Tg(Alb-cre)21Mgn/J	JAX: 003574
2014	Stewart et al., 2014 (28)	HCS	-	Ghrfl/fl;Vav1Cre/+	LeRoith	Tg(Vav1-cre)1Awr	MGI ID: 3043860
2015	Gesing et al., 2015 (11)	Liver	-	LiGHRKO	Kopchick	B6.Cg-Tg(alb-cre)21Mgn/J	JAX: 003574
2015	Dominick et al., 2015 (12)	Liver	-	LiGHRKO	Kopchick	B6.Cg-Tg(alb-cre)21Mgn/J	JAX: 003574
2015	Zawada et al., 2015 (13)	Liver	-	LiGHRKO	Kopchick	B6.Cg-Tg(alb-cre)21Mgn/J	JAX: 003574
2015	List et al., 2015 (37)	Muscle	-	MuGHRKO	Kopchick	B6.FVB(129S4)-Tg(Ckmm-cre)5Khn/J	JAX: 006475
2015	Cordoba-Chacon et al., 2015 (15)	Liver	Yes	aLivGHRkd	Kopchick	Tail vein injection of Adeno-associated virus-TBGp-Cre	Penn Vector Core: AV-8-PV1091
2015	Sadagurski et al., 2015 (14)	Liver	-	LiGHRKO	Kopchick	B6.Cg-Tg(alb-cre)21Mgn/J	JAX: 003574
2016	Liu et al., 2016 (29)	Bone	-	DMP-GHRKO	LeRoith	B6.Cg-Tg(Dmp1-cre/ERT2)D77Pdp/J^†^	JAX: 029594
2016	Jara et al., 2016 (30)	Heart	Yes	iC-GHRKO	Kopchick	B6.FVB(129)-A1cfTg(Myh6-cre/Esr1*)1Jmk/J	JAX: 005657
2016	Liu et al., 2016 (16)	Liver	-	Li-GHRKO	LeRoith	B6.FVB(129)-Tg(Alb1-cre)1Dlr/J	JAX: 016832
2016	Junilla et al., 2016 (32)	Global	Yes	aGHRKO	Kopchick	B6.129-Gt(ROSA)26Sortm1(cre/ERT2)Tyj/J	JAX: 008463
2017	Cady et al., 2017 (40)	Brain	-	LeprEYFP∆GHR	Kopchick	B6.129-Leprtm3(cre)Mgmj/J	JAX: 032457
2019	List et al., 2019 (20)	Fat	-	AdGHRKO	Kopchick	B6;FVB-Tg(Adipoq-cre)1Evdr/J	JAX: 010803
2019	Fang et al., 2019 (7)	Liver	-	Fat-Ghr-/-	Liang	B6;FVB-Tg(Adipoq-cre)1Evdr/J	JAX: 010803
2019		Fat	-	L-Ghr-/-	Liang	B6.CgTg(Alb-cre)21Mgn/J	JAX: 003574
2019	Furigo et al., 2019 (41)	Brain	-	AgRP GHR KO	Kopchick	Agrptm1(cre)Lowl/J	JAX: 012899
2019		Brain	-	LepR GHR KO	Kopchick	B6.129-Leprtm2(cre)Rck/J	JAX: 008320
2019		Brain	-	Brain GHR KO	Kopchick	B6.Cg-Tg(Nes-cre)1Kln/J	JAX: 003771
2019	Young et al., 2019 (31)	Intestine	-	IntGHRKO	Kopchick	B6.Cg-Tg(Vil1-cre)997Gum/J	JAX: 004586

† The name was changed from GHRMacD to MacGHR KO in this paper.

## DISRUPTION OF GHR IN LIVER

### History

Liver is the most targeted tissue ( 11 out of 28 ) in conditional GHRKO studies with 5 distinct liver-specific GHR knockout mouse lines generated. The first mouse line, termed GHR liver deficient (GHRLD), was produced at the University of Pittsburgh by Fan and cols. in 2009 and represents the first of any reported tissue-specific GHRKO mouse lines. This line was generated using the GHR floxed mice from the Sperling laboratory and was crossed with albumin Cre mice (B6.Cg-Tg(Alb-cre)21Mgn/J, Stock# 003574, The Jackson Laboratory). This line has been used in two published studies ( 4 , 8 ). The second was produced at Ohio University by List and cols. in 2013 and is termed the liver-specific GHR knockout mouse (LiGHRKO). This mouse line was generated by crossing the Kopchick floxed GHR mice with albumin Cre mice (B6.Cg-Tg(Alb-cre)21Mgn/J, Stock# 003574, The Jackson Laboratory) and has been used in six studies ( 9 - 14 ). The third liver-specific line was generated at the University of Illinois by Cordoba-Chacon and cols. in 2015 and is termed the adult-onset, liver-specific GHR knockdown mouse (aLivGHRkd). The aLivGHRkd mice were produced using Kopchick floxed by injecting GHR floxed mice with an adeno-associated virus carrying a liver-specific thyroxine-binding globulin promoter (AAV8.TBG.PI.Cre.rBG, Penn Vector Core). This mouse line has been used in one study ( 15 ). The fourth line was generated at the NYU College of Dentistry by Liu and cols. in 2016 and is termed the Li-GHRKO mouse. This line was produced using LeRoith floxed GHR mice crossed with albumin Cre mice (B6.Cg-Tg(Alb-cre)21Mgn/J, Stock# 003574, The Jackson Laboratory) and has been used in one study ( 16 ). The fifth liver-specific mouse line was generated at the University of Texas by Fang and cols. in 2019. These mice were termed L-Ghr-/- and were produced by crossing Liang floxed GHR mice to albumin/Cre mice (B6.Cg-Tg(Alb-cre)21Mgn/J, Stock# 003574, The Jackson Laboratory) and has been used in one study ( 7 ).

### Results

Phenotypic changes in the various liver-specific GHRKO lines are numerous and severe. As expected, disruption of GHR in liver results in multiple changes to the GH/IGF-1 axis, including decreased serum levels of IGF-1, ALS, IGFBP-3, -5 and 7 and increased GH, IGFBP-1 and -2 levels ( 4 , 9 , 16 ). Despite dramatic reductions to circulating IGF-1, elevated GH resulted in elevated local IGF-1 mRNA expression in skeletal muscle, brown adipose tissue, and white adipose tissue (subcutaneous and retroperitoneal depots) ( 9 ). Evaluation of pituitary showed increased GH, GH releasing-hormone receptor, and ghrelin receptor mRNA ( 7 ). Measures of glucose homeostasis are substantially altered in these mice as they have elevated blood glucose, insulin and C-peptide levels, impaired glucose tolerance, and reduced insulin sensitivity ( 4 , 9 , 16 ). However, when placed on caloric restriction, Fang and cols., report greater hypoglycemia compared to controls ( 7 ). Liver specific disruption also causes substantial changes to serum lipids and cholesterol as these mice have an increase in circulating free fatty acids, triglycerides, cholesterol, VLDL, and LDL ( 4 , 8 , 16 ). Importantly, several laboratories have shown that these mice develop fatty liver ( 4 , 9 , 15 , 16 ). However, when calorically restricted, Fang and cols report a greater decrease in liver triglycerides compared to controls ( 7 ). This group also showed that the fatty livers progress to hepatic fibrosis with increased fibrotic marker, namely, Col1A2 and Col3A1 mRNA ( 8 ). Studies by two separate laboratories indicate increased inflammation ( 4 , 9 ), specifically through upregulation of TNF-α ( 8 ). Additional cytokines have been measured and shown to be elevated. Increased MCP-1, IL-6 ( 9 ), TGF-β ( 8 ), as well as an increase in liver mRNA expression of TNF-α, IL-6, IL-18, and IL-1β ( 16 ) have been reported. In addition to inflammatory cytokines, several adipokines are increased, including resistin, adiponectin, leptin, and adipsin ( 9 , 16 ). Measures of oxidative stress (SOD, GPX, and Nrf 2 ) are increased in livers of these mice ( 16 ). Cordoba and cols. describe increase in liver de novo lipogenesis, as well as an increase in glycolysis, determined through an increase in fructose 2,6-bisphosphate and glucokinase ( 15 ). Fang and cols. discovered that L-Ghr-/- mice had a significant reduction in liver autophagic vacuoles ( 7 ), and Fan and cols. reported decreased bone density in GHRLD mice ( 4 ). Body weight and length are unchanged in early life, but in older mice (after 5 months of age), both of these measures are significantly decreased in both sexes ( 9 ). Furthermore, analysis of body composition over time by List and colleagues revealed a body composition pattern similar to those found in GH transgenic mice ( 17 ); that is, LiGHRKO mice had a higher percentage of body fat at early ages followed by lower percentage of body fat in adulthood. Lastly, despite dramatic changes to liver as well as whole body physiology, disruption of GHR in liver does not alter lifespan ( 12 ).

## DISRUPTION OF GHR IN ADIPOSE TISSUE

### History

Three distinct adipose-specific lines have been reported. The first was generated at Ohio University by List and cols in 2013 and is referred to as the FaGHRKO mouse ( 6 ). The mouse was generated by crossing the Kopchick floxed GHR mouse line to aP2 (also called Fabp 4 ) Cre expressing mice (B6.Cg-Tg(Fabp4-cre)1Rev/J, Stock#005069, The Jackson Laboratories). Importantly, the aP2-cre promoter/enhancer has more recently been shown to have expression in non-adipose tissues or cells ( 18 , 19 ), which makes it challenging to interpret the results in the context of solely impacting adipose tissue, and was the impetus for generation of the second fat specific GHRKO mouse line from the Kopchick laboratory. This second mouse line was also produced at Ohio University by List and cols., in early 2019 and is termed AdGHRKO ( 20 ). The mouse was generated by crossing the Kopchick floxed GHR mouse line with adiponectin Cre expressing mice (B6;FVB-Tg(Adipoq-cre)1Evdr/J, Stock#006475, The Jackson Laboratories). The authors refer to this mouse strain as “adipocyte” specific as opposed to targeting adipose tissue due to the higher fidelity of the adiponectin promoter, which is considered exclusive to mature adipocytes. The third mouse line was generated at the University of Texas by Fang and cols. in 2019 ( 7 ). These mice were termed Fat-Ghr-/- and were produced by crossing Liang floxed GHR mice to adiponectin Cre expressing mice (B6;FVB-Tg(Adipoq-cre)1Evdr/J, Stock#006475, The Jackson Laboratories).

### Results

As might be expected, all three lines exhibit loss of GHR mRNA in all white and brown adipose depots. Notably, the Fat-Ghr-/- mice also have a small but significant decrease in GHR mRNA in the heart, which the authors attribute to residual epicardial and pericardial fat; this difference is not evident in the hearts of AdGHRKO mice that utilize the same Cre line ( 20 ). In terms of phenotype, very little data are reported for the Fat-GHR-/- mice as the major focus of this paper was the liver specific GHR (L-GHR-/-) mice described above. The only data reported are measurements of fat mass as well as circulating levels of blood glucose, ghrelin, and GH for male mice that were 7-9 weeks old, which were calorie restricted for 11 days. No difference between controls and Fat-GHR-/- mice were found.

More comprehensive and comparable analyses were performed for the FaGHRKO and AdGHRKO mice. Both studies used male and female mice and with measurements taken up to six months of age ( 6 , 20 ). Overall, these two lines have a similar adipose tissue profile. Both FaGHRKO and AdGHRKO mice have increased total fat mass with the most dramatic increase in the subcutaneous depot as has been reported for global GHRKO mice ( 21 , 22 ). As for the other depots, the mass of all are significantly enlarged in the tissue specific lines relative to controls, except male perigonadal in FaGHRKO mice ( 6 ) and male perigonadal and male BAT in AdGHRKO mice ( 20 ). Other features of white adipose tissue reported for these two tissue specific lines includes enlarged adipocyte size and reduced collagen deposition in white fat ( 23 ), with the difference more pronounced in the subcutaneous depot. Thus, the pattern of fat deposition and the basic characteristics of adipose tissue are strikingly similar between the two tissue-specific mouse lines despite using different promoter/enhancers to drive Cre expression. As for adipokines, both sexes of AdGHRKO mice have significantly decreased levels of resistin and adiponectin while FaGHRKO mice have increased leptin, no change in resistin, and decreased adiponectin in only males ( 6 , 20 , 24 ). Thus, despite no change in overall mass and morphology, there are molecular differences in adipose tissue between AdGHRKO and FaGHRKO mice. There are other important differences in several metabolic parameters and in growth between FaGHRKO and AdGHRKO mice. First, FaGHRKO mice weigh more and have an increase in body length compared to controls, but AdGHRKO mice have no significant difference in weight or length relative to controls. This is likely due to FaGHRKO mice having elevated circulating IGF-1, at least in males, and a trend for increased GH in both sexes while AdGHRKO mice have no changes in GH or IGF-1 levels. There are also differences in glucose metabolism. AdGHRKO mice have decreased circulating insulin and enhanced insulin sensitivity, which is consistent with global GHRKO mice ( 2 ) while both male and female FaGHRKO mice exhibit no alteration in measures of glucose homeostasis. Finally, both male and female AdGHRKO mice have reduced liver triglycerides, which is distinct from what is observed for liver triglycerides in FaGHRKO mice (no change) and in the global GHRKO mice (no change or increased depending on age) ( 22 ). Due to improved glucose homeostasis in AdGHRKO mice the authors suggest that the AdGHRKO mice, with a more specific disruption of GH in adipocytes, have an overall healthier metabolic profile than FaGHRKO mice.

## DISRUPTION OF GHR IN MACROPHAGES

### History

Two papers from Ram Menon’s laboratory at University of Michigan report characterization of a macrophage specific GHR disrupted mouse line ( 25 , 26 ). Both papers utilize the same strain of mice with the initial publication coming in 2010. These mice were created by breeding Sperling floxed GHR mice to mice that express Cre recombinase from the endogenous Lyzs locus (B6.129P2-Lyz2tm1(cre)Ifo/J, Stock#004781, The Jackson Laboratory). Importantly, this Cre recombinase, in addition to targeting macrophages, is also expressed in monocytes and granulocytes ( 27 ). Despite the same mice, the authors use two different designations to describe them: GHRMacD in the 2010 publication and GHRMac KO in the 2013 manuscript.

### Results

As expected, GHR expression is greatly diminished in macrophages isolated from either adipose tissue or from the peritoneal cavity and in isolated monocytes but without alteration of GHR expression in whole adipose tissue or liver. The first paper did not report general features of GHRMacD mice ( 25 ). Instead, this paper only reports data for isolated primary macrophages from these mice for *in vitro* studies. Specifically, they report that conditioned media from adipose tissue-derived GHRMacD macrophages exhibit a greater inhibitory effect on 3T3-L1 preadipocyte differentiation and adipogenesis compared with conditioned media from macrophages of control mice. These results indicate that intact GH action in adipose-derived macrophages is important for preadipocyte differentiation. Further, they show that the increase in adipogenesis is not mediated by IGF-1. The subsequent paper provides a more thorough characterization of the mouse ( 26 ). On a standard chow diet, GHRMac KO mice are indistinguishable from control mice with respect to weight and glucose homeostasis. However, when fed a high fat diet for 18 weeks, differences between GHRMac KO mice and controls emerge. With high fat diet feeding, GHRMac KO mice have impaired glucose homeostasis as well as an increase in specifically epididymal fat mass, despite no change in whole body composition measurements. In addition, the epididymal fat pad exhibits enlarged adipocytes, an increase in stromal vascular (SVF)-derived macrophages (most notably the classically activated M 1 macrophages), increased inflammation (determined by both expression of pro-inflammatory cytokines and histologically, by crown like structures), and increased expression of osteopontin from the SVF via a NF-κB site in the distal osteopontin promoter. Overall, their data suggest that a lack of GH action in macrophages results in a deterioration in glucose metabolism by promoting enhanced adipose tissue inflammation during obesity. These data led the authors to hypothesize that GH treatment could promote a beneficial effect on the chronic inflammation and insulin resistance observed in obesity.

## DISRUPTION OF GHR IN PANCREATIC BETA CELLS

### History

Pancreatic b-cell specific GHRKO mice (bGHRKO) were produced by Wu and cols in 2011 at Mount Sinai School of Medicine. This mouse line was generated using the LeRoith GHR floxed mice crossed with the rat insulin II promoter (RIP)-driven Cre recombinase mice (B6.Cg-Tg(Ins2-cre)25Mgn/J, Stock# 003573, The Jackson Laboratories).

### Results

Phenotypic changes are mild in the bGHRKO mouse line, with the only alteration seen in glucose homeostasis. That is, disruption of GHR in b cells does not affect growth or development (as measured by body weight, composition, and IGF- 1 levels) nor did it affect insulin development as seen by insulin levels, islet size, or insulin content in pancreatic islets ( 5 ). The first phase of insulin secretion is blunted in the βGHRKO mouse line, suggesting lower maximal insulin secretion when challenged with a high glucose load. When placed on a high fat diet, obese βGHRKO mice display impaired glucose homeostasis and β cell hyperplasia with decreased β cell proliferation and total β cell mass, suggesting that GH plays an important compensatory role in β cells in response to a high glucose challenge.

## DISRUPTION OF GHR IN HEMATOPOIETIC STEM CELLS (HSC)

### History

HSC specific GHRKO mice (GHR^fl/fl;Val1Cre/+^) were produced by Stewart and cols. in 2014 at Boston Children’s Hospital ( 28 ). This line was generated by crossing the LeRoith GHR floxed mice with the Vav1 Cre transgenic mice (Tg(Vav 1 -cre)1Awr, MGI:304860).

### Results

The GHR^fl/fl;Val1Cre/+^ mice show no difference compared to controls in steady-state hematopoiesis, reconstitution, lineage potential, self-renewal, or HSC activity upon serial transplantation or 5-fluorouracil challenge ( 28 ). Thus, the study concluded that ablation of the GHR in HSC is dispensable for HSC activation and recovery.

## DISRUPTION OF GHR IN BONE

### History

The dentin matrix protein (DMP)-1 mediated GHRKO (DMP-GHRKO) mouse line was produced by Liu and cols. at New York University College of Dentistry in 2016 ( 29 ). This mouse line was generated by crossing LeRoith GHR floxed mice with DMP-1 promoter Cre recombinase mice (B6.Cg-Tg(Dmp1-cre/ERT2)D77Pdp/J, Stock# 029594, The Jackson Laboratories), which is specific for mature osteoblasts and osteocytes.

### Results

Phenotypic changes observed in the DMP-GHRKO mice are specific to bone acquisition. These mice did not have altered body weight, body composition, linear growth or serum IGF-1 levels ( 29 ). DMP-GHRKO mice have a slender bone phenotype with decreased total cross-sectional area, bone tissue at the femur mid-diaphysis, bone formation rate, mineral apposition rate, and number of osteoblasts, and increased number of osteoclasts. Moreover, these mice have impaired inorganic phosphate homeostasis and decreased serum parathyroid hormone levels with an impaired response to intermittent, anabolic parathyroid hormone treatment. This study concluded that GH action in bone – along with that of parathyroid hormone – is necessary for bone accrual, cell viability, matrix mineralization, and inorganic phosphate homeostasis.

## DISRUPTION OF GHR IN HEART

### History

Inducible cardiac-specific GHR disrupted (iC-GHRKO) mice were produced by Jara and cols. in 2016 at Ohio University ( 30 ). The iC-GHRKO mouse line was generated by crossing Kopchick GHR floxed mice with myosin heavy chain (Myh 6 )-driven MerCreMer (MCM) recombinase mice (B6.FVB(129)-A1cfTg(Myh6-cre/Esr1*)1Jmk/J, Stock#005657, The Jackson Laboratories). As an inducible cre system, disruption of cardiac GHR was induced by tamoxifen at four months of age.

### Results

Phenotypic changes in the iC-GHRKO mouse line are modest and age-dependent, specifically related to body composition, metabolism, and glucose homeostasis. The iC-GHRKO mice experience a shift in body composition (decrease in fat mass and increase in lean mass) in early adulthood (4.5 to 8.5 months of age) and improved insulin sensitivity by 6.5 months ( 30 ). By 12.5 months, iC-GHRKO mice no longer display significant decreases in fat mass and develop impaired glucose homeostasis with significantly decreased insulin stimulated Akt phosphorylation in the heart and liver and decreased circulating IGF-1 levels. Surprisingly, disruption of cardiac GHR did not affect the cardiac phenotype with no change in heart size, maintenance of baseline or dobutamine-stressed echocardiography, or systolic blood pressure. Surprisingly, these results suggest that adult GHR disruption in cardiomyocytes is crucial in the regulation of systemic metabolic homeostasis but not cardiac function.

## DISRUPTION OF GHR IN INTESTINES

### History

Intestinal epithelial cell-specific GHRKO (IntGHRKO) mice were produced by Young and cols. in 2019 at Ohio University ( 31 ). This line was generated by crossing Kopchick GHR floxed mice with villin promoter/enhancer-driven Cre recombinase mice (B6.Cg-Tg(Vill-cre)997Gum/J, Stock#004586, The Jackson Laboratories).

### Results

IntGHRKO mice exhibit modest, sex-specific changes to the intestinal phenotype (i.e. gross anatomy, gut barrier, and fat absorption) and glucose homeostasis ( 31 ). Male IntGHRKO mice have significantly decreased large intestinal length and impaired fat absorption. Only female IntGHRKO mice displayed significant glucose intolerance and insulin resistance. Both sexes of IntGHRKO mice showed a weak improvement to gut barrier function. Overall, this study suggests that disruption of GHR in the intestinal epithelial cells has a mild impact on intestinal growth and function.

## ADULT-ONSET DISRUPTION OF GLOBAL GHR

### History

Adult-onset GHRKO (aGHRKO) mice were produced by Junnila and cols. in 2016 at Ohio University ( 32 ). This mouse line was generated by crossing Kopchick GHR floxed mice with ROSA26 gene promotor/enhancer-driven Cre recombinase mice (B6.129-Gt(ROSA)26Sortm1(cre/ERT2)Tyj/J, Stock#008463, The Jackson Laboratories), and disruption of the *Ghr* gene was induced through tamoxifen at six weeks of age.

### Results

The aGHRKO mice share many phenotypic changes with the original global GHRKO mice ( 32 ). That is, the aGHRKO mice have reduced body length and weight with increased adiposity, specifically in subcutaneous adipose tissue, impaired glucose tolerance, and improved insulin sensitivity. The aGHRKO mice also have increased adiponectin and resistin levels. Moreover, aGHRKO mice display increased maximal lifespan compared to controls, although this finding was unique to females. These findings show that disruption of GHR in adulthood can recapitulate the beneficial metabolic and longevity findings – at least in females – seen with the global GHRKO mice.

## DISRUPTION OF GHR IN MUSCLE

### History

To date, three distinct muscle specific GHR knockout mouse lines have been reported. The first line was generated at John Hopkins University School of Medicine by Mavalli and cols. in 2010 and is referred to as ΔGHR mice ( 33 ). The mouse line was generated by crossing Sperling floxed GHR mice with mef-2c-73k promoter Cre mice (Mef-2c-73kCre+/-, Brian Black, University of California San Francisco). The second mouse line was generated at Mount Sinai School of Medicine by Vijaykumar and cols in 2012 and is referred to as mGHRKO mouse ( 34 ). This mouse line was generated by crossing the LeRoith floxed GHR mice with muscle creatine kinase Cre mice (B6.FVB(129S4)-Tg(Ckmm-cre)5Khn/J, Stock# 006475, The Jackson Laboratories). This mouse line has been used in three studies ( 34 - 36 ). The third mouse line was generated at Ohio University by List and cols. in 2015 and is referred to as MuGHRKO mouse ( 37 ). The mouse line was generated by crossing the Kopchick floxed GHR mice with muscle creatine kinase Cre mice (B6.FVB(129S4)-Tg(Ckmm-cre)5Khn/J (Stock# 006475, The Jackson Laboratories).

### Results

The ΔGHR mice, which used a distinct Cre line, have reduced myofiber number and area with deficiencies in muscle performance ( 33 ). The ΔGHR mice also have diminished myoblast fusion, peripheral adiposity, glucose intolerance, and insulin resistance ( 33 ). These results are in stark contrast to the other two muscle specific lines, mGHRKO and MuGHRKO. Importantly, the promoter/enhancer used to drive Cre-recombinase to generate ΔGHR mice (mef-2c-73k) has been shown to be involved in the regulatory processes of brain, neural crest, bone, craniofacial, melanocyte, lymphocyte, endothelium, and blood vessel development ( 38 , 39 ), making interpretations of data generated in ΔGHR mice challenging. The two other muscle specific GHRKO mouse lines (mGHRKO and MuGHRKO) show marked health improvements. Male MuGHRKO mice have reduced levels of glucose, insulin, c‐peptide, and enhanced glucose tolerance ( 37 ), while mGHRKO mice on a high fat diet have decreased adiposity, inflammation, muscle and hepatic triglyceride content, and enhanced insulin sensitivity ( 34 ). High fat fed mGHRKO mice have greater energy expenditure with increased respiratory exchange ratios, suggesting increased carbohydrate utilization ( 34 ). Muscle performance, treadmill endurance, and grip strength are unchanged with advanced age in MuGHRKO mice, leading the authors to suggest that the direct action of GH on muscle has minimal effect on strength or endurance ( 37 ). Importantly, disruption of GHR in muscle is shown to increase lifespan in male MuGHRKO mice ( 37 ).

## DISRUPTION OF GHR IN BRAIN

### History

Four distinct neuronal specific GHR knockout mice have been reported. The first line was generated at University of Michigan by Cady and cols. in 2017 ( 40 ) with ablation of GHR in leptin receptor (LepRb)-expressing neurons and is referred to as Lepr^EYFPΔGHR^. The mouse line was generated by crossing the Kopchick floxed GHR mice with Lepr^cre^ mice (B6.129-Leprtm3(cre)Mgmj/J, Stock# 032457, The Jackson Laboratories). The next three mouse lines were generated at University of São Paulo by Furigo and cols. in 2019 ( 41 ). All three lines used the Kopchick floxed GHR line crossed with the following Cre lines: AgRP-IRES-Cre mouse (Agrp^tm1(cre)Lowl^/J, The Jackson Laboratory) to produce AgRP GHR KO mice, leptin receptor LepR-IRES-Cre mouse (B6.129-Lepr^tm2(cre)Rck^/J he Jackson Laboratory) to produce LepR GHR KO mice, and Nestin-Cre mouse (B6.Cg-Tg^(Nes-cre)1Kln^/J, The Jackson Laboratory) to produce Brain GHR KO mice.

### Results

Increased body weight and length are reported in LepR GHR KO and brain GHR KO mice ( 41 ). Interestingly, during caloric restriction, a higher rate of weight loss is exhibited by AgRP GHR KO, LepR GHR KO, and brain GHR KO mice ( 41 ). Lepr^EYFPΔGH^ mice on both chow and HFD are glucose intolerant with normal insulin tolerance compared to controls ( 40 ). In clamp studies, Lepr^EYFPΔGH^ mice show a significant reduction in glucose infusion rate, which suggests that these mice cannot maintain euglycemic conditions, and exhibit significantly higher hepatic glucose production ( 40 ). Additionally, hepatic insulin resistance is confirmed with attenuation of insulin stimulated phosphorylation of IRD-1 and Akt (Ser 473) in the liver of Lepr^EYFPΔGH^ as compared to controls. In contrast, glucose tolerance and insulin sensitivity were unchanged in AgRP GHR KO as compared to controls ( 41 ). However, during the initial days of caloric restriction, AgRP GHR KO exhibit lower glycemia when compared to controls in both males and females ( 41 ). Lower glycemia is also exhibited by LepR GHR KO. Overall, ablation of GHR in LepRb neurons disrupts glucose homeostasis and energy conservation by AgRP neurons during caloric restriction ( 41 ).
